# Elevated resting heart rate is a marker of subclinical left ventricular dysfunction in hodgkin lymphoma survivors

**DOI:** 10.1016/j.ijcha.2021.100830

**Published:** 2021-06-25

**Authors:** Julius C. Heemelaar, Augustinus D.G. Krol, Marloes Louwerens, Saskia L.M.A. Beeres, Eduard R. Holman, Martin J. Schalij, M. Louisa Antoni

**Affiliations:** a Department of Cardiology, Heart Lung Center, Leiden University Medical Centre, Leiden, the Netherlands; b Department of Radiotherapy, Leiden University Medical Centre, Leiden, the Netherlands; c Department of Internal Medicine, Leiden University Medical Centre, Leiden, the Netherlands; d Department of Cardiology, Heart Lung Center, Leiden University Medical Centre, Leiden, the Netherlands; e Department of Cardiology, Heart Lung Center, Leiden University Medical Centre, Leiden, the Netherlands; f Department of Cardiology, Heart Lung Center, Leiden University Medical Centre, Leiden, the Netherlands; g Department of Cardiology, Heart Lung Center, Leiden University Medical Centre, Leiden, the Netherlands

**Keywords:** CARDIO-ONCOLOGY, HODGKIN LYMPHOMA, HEART FAILURE, RESTING HEART RATE, ECHOCARDIOGRAPHY, THORACIC IRRADIATION

## Abstract

**Background:**

Thoracic irradiation is one of the cornerstones of Hodgkin lymphoma (HL) treatment, which contributes to high rates of long-term survivorship, but begets a life-long increased risk of heart disease including heart failure. At the cardio-oncology (CO) clinic, persistent sinus tachycardia or elevated resting heart rate (RHR) is frequently observed in these patients. The aim of this study was to evaluate the relation between RHR and left ventricular (LV) dysfunction.

**Methods:**

In 75 HL survivors visiting our CO-clinic echocardiographic evaluation of LV systolic and diastolic function including global longitudinal strain (GLS) was performed to assess subclinical LV dysfunction.

**Results:**

Median age of HL diagnosis was 24 [25th-75th percentile: [19], [29]] years with a 17 [12], [25] year interval to CO-clinic visit and 31 patients (41%) were male. Average RHR was 78 ± 14 bpm and 40% of patients (N = 30) had an elevated RHR defined as ≥ 80 bpm. While there was no difference in LV ejection fraction (55.6 ± 4.3 vs*.* 54.8 ± 6.6; *p* = 0.543), patients with elevated RHR had abnormal GLS (-15.9% vs*.* −18.3%, *p* = 0.045) and higher prevalence of diastolic dysfunction (73.3% vs*.* 46.7%; *p* = 0.022). GLS, E/e’ ratio and presence of diastolic dysfunction were independently associated with RHR when correcting for age, sex and mantle field irradiation. A significant improvement was observed of the RHR-association model with solely extracardiac confounders when LV-function parameters were added to the model (F-statistic = 6.36, *p* = 0.003).

**Conclusions:**

This study indicates RHR as a possible marker for subclinical LV-dysfunction in HL survivors.

## 1 Introduction

The successful implementation of thoracic irradiation and anthracycline-containing chemotherapy regimens in Hodgkin lymphoma (HL) - one the most prevalent malignancies in adolescents and young adults - led to a long term survival of above 80% [1]. The reverse of the medal is that these treatment modalities beget a life-long increased risk of a multitude of cardiovascular disease in HL survivors compared to the general population [2], [3], [4].

To mitigate this risk – as recommended by international guidelines - specialised cardio-oncology (CO) clinics for the detection of late cardiovascular side effects of cancer treatment have been established [5], [6], [7].

Frequently HL survivors who received thoracic irradiation show persistent sinus tachycardia or an elevated resting heart rate (RHR) [8], [9]. RHR over the years is a thoroughly studied research topic and is identified as an important prognostic factor in the general population [10], [11] and in cardiovascular disease patients [12], [13], [14]. Custodis and colleagues accurately depict RHR as a central factor that influences all stages of cardiovascular disease: from risk factor, to the occurrence of adverse cardiac events and heart failure. An elevated RHR in cancer patients predicts increased mortality rates [9], [15].

Most research regarding elevated RHR in cancer survivors focusses on autonomic dysfunction caused by thoracic irradiation [16], [17]. Groarke and colleagues also displayed prognostic implications of the presence of autonomic dysfunction in these patients. However, there is limited knowledge about the relation between left ventricular (LV) dysfunction and elevated RHR in patients after cancer treatment.

Several studies have already demonstrated that cancer survivors whom underwent thoracic irradiation or anthracycline treatment often show echocardiographic evidence of LV dysfunction [18], [19], [20], [21]. Also, in the early stages of heart failure increased adrenergic activity is a well-known phenomenon [22].

Therefore, we hypothesised that there may be a relation between the presence of an elevated resting RHR and LV dysfunction in HL survivors whom underwent thoracic irradiation and evaluated echocardiographic parameters of systolic and diastolic function in these patients presenting at the CO-clinic.

## 2 Methods

**Fig. 1:**
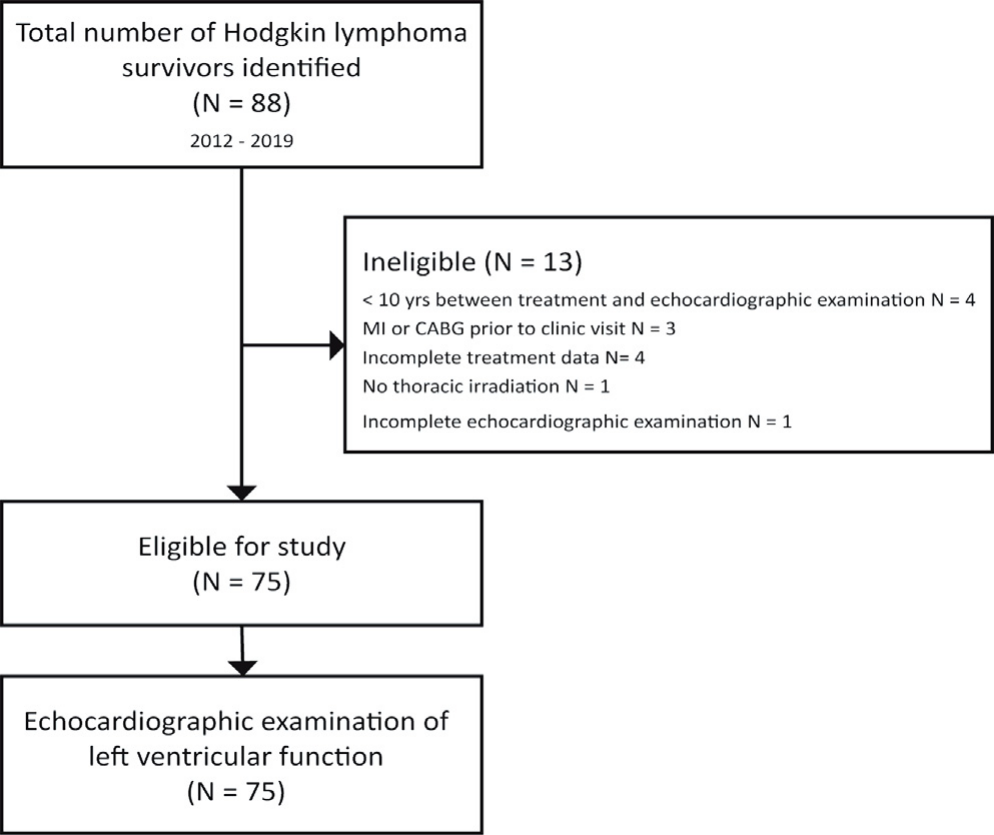
Selection process of the study population. MI = myocardial infarction, CABG = coronary artery bypass grafting,

A retrospective cohort study among HL survivors treated with mediastinal irradiation referred to the cardio-oncology outpatient clinic at the Leiden University Medical Centre was performed. Patients are referred to the CO-clinic through the national HL survivorship care pathway, where all HL survivors between 15 and 60 years of age are eligible for routine cardiovascular screening. Between 2012 and 2019 88 unique HL survivors were identified (Fig. 1*)*. All patients underwent transthoracic echocardiographic examination according to clinical protocol. Patients were eligible for inclusion in the study if they (i) were diagnosed with a histologically confirmed HL, and (ii) received thoracic radiotherapy at least ten years prior to referral to the CO-clinic. For the purpose of this study patients with a history of myocardial infarction or cardiac surgery prior to echocardiographic examination were excluded. Furthermore, patients were excluded in case of incomplete HL diagnosis- and treatment history or incomplete echocardiographic examination. Also patients with persistent atrial fibrillation, atrial flutter or supraventricular tachycardia during echocardiographic examination were excluded from this study. The local Medical Ethical Committee approved this study. All patients gave informed consent to participate in the study. This study complies with the Declaration of Helsinki.

When patients were eligible for inclusion, medical records were reviewed to acquire detailed HL diagnosis and treatment data including Ann Arbor stage, radiotherapy treatment plan, cumulative mediastinal radiation dose, chemotherapy regimen and cumulative dose of anthracyclines. Additionally, demographic characteristics, traditional cardiovascular risk factors and medications at time of index clinic visit were collected from the departmental Cardiology Information System (EPD-Vision®, Leiden University Medical Centre, Leiden, the Netherlands).

Hypercholesteremia included any history of hypercholesteremia or statin use at index clinic visit. Family history of heart disease was defined as one or more first-degree relatives with myocardial infarction, cardiac surgery or sudden cardiac death before 60 years of age. Chronic renal insufficiency was defined as an estimated glomerular filtration rate below 60 mL/min/1.73 m^2^ using the CKD-EPI formula.

RHR was determined during echocardiographic examination after at least ten minutes of supine rest when a stable RHR was established. An elevated RHR was defined as sinus rhythm equal or >80 beats per minute (bpm). The latter cut-off was chosen in line with previous publications regarding elevated RHR and excess mortality risk [13], [16].

Patients were imaged in the left lateral decubitus position using the Vivid 7, E9 and E95 systems (General Electric Healthcare Vingmed, Horten, Norway). Data acquisition was performed with 3.5 MHz or M5S transducers. Standard 2D, color, pulsed wave and continues wave Doppler images were acquired and stored for offline analysis (Echopac BT13; GE Medical Systems, Milwaukee, WI).

LV end-systolic, end-diastolic volume indexes (LVESVi, LVEDVi) were quantified from the apical two- and four-chamber view using the modified Simpson rule, and subsequently the LV ejection fraction (LVEF) was derived [23]. Left atrial volume index (LAVi) was calculated using Simpson biplane method.

LV diastolic function was quantified using mitral inflow Doppler E- and A-wave velocities, E/A ratio and E-wave deceleration time measurement. Tissue Doppler imaging was performed to measure peak early diastolic tissue velocity from the septal and lateral mitral annulus (e’-wave) and to calculate E/e’ ratio. Diastolic function was graded by American Society of Echocardiography (ASE) and European Association of Cardiovascular Imaging (EACVI) recommendations [24].

Two-dimensional speckle-tracking strain echocardiography was applied to evaluate LV global longitudinal strain (GLS) using commercially available software (EchoPAC version 203). This technique quantifies myocardial deformation by tracking myocardial acoustic markers in every frame within the cardiac cycle. The LV endocardial border was traced in apical three-, four- and two-chamber views and the region of interest encompassing the LV myocardial wall was adjusted manually where needed. Calculation of the LV GLS is performed by the software as the average of the longitudinal strain values of each apical view and a color-coded 17-segment bulls-eye plot is provided. GLS is expressed as a negative value reflecting the shortening of the LV myocardium, thus more negative value represents better deformation. Systolic dysfunction was defined as LVEF < 50% or GLS > -16% [25], [26]. Any grade of diastolic dysfunction was defined as presence of at least 2 of the following criteria: E/e’ >14, septal e’ <7 or lateral e’ <10, TR velocity > 2.8 or LAVi > 34 [24].

Continuous variables are presented as mean ± SD, or median with 25th and 75th percentile depending on variable-distribution. Normality of distribution was assessed graphically and by Kolmogorov-Smirnov test. Categorical data are presented as frequencies and percentages.

Differences in baseline characteristics and echocardiographic parameters between patients with- and without elevated RHR were evaluated using unpaired Student’s *t*-test, Mann-Whitney *U* test, Chi-square or Fisher’s exact test, where appropriate.

Both simple- and explanatory multilinear regression modeling was performed to evaluate the association of echocardiographic parameters to RHR as a continuous variable. Possible confounders were identified by literature review added to the multilinear model: age, sex, mantle field irradiation.

Furthermore, an F-test for nested regression model comparison was used to compare a model restricted to extracardiac confounders (age + sex + mantle field irradiation) - and an unrestricted model with both extracardiac confounders and markers of cardiac function in relation to RHR.

Missing data was accounted for using complete-case analysis. Imputation methods for missing echocardiographic measurements were not contemplated, because they were assumed to be Missing Not At Random.

All statistical analyses were performed in R version 3.6.3 (R Foundation for Statistical Computing, Vienna, Austria).

## 3 Results

A total of 75 patients were enrolled in the study. The selection process is shown in Fig. 1. Baseline characteristics of the study population are shown in Table 1. The median age of HL diagnosis was 24 [19.0–29.5] years with an interval of 17.6 [12.4–25.8] years to index CO-clinic visit. The median cumulative mediastinal radiation dose was 36 [25th - 75th percentile: [35], [36], [37], [38], [39], [40]] Gy, and the majority of patients received concomitant anthracycline-containing chemotherapy regimens (N = 54, 72%). Only few patients used medication at index CO clinic visit with 6 patients (8%) using betablockers. Sixty patients (80%) did not use any medication. All patients had sinus rhythm at index CO-clinic visit.

**Table 1:** Baseline characteristics of study population.

Variable	Overall	Heart rate < 80 bpm.	Heart rate ≥ 80 bpm.	
	**(N = 75)**	**(N = 45)**	**(N = 30)**	***p*-value**
Resting heart rate, bpm	77 ± 14	69 ± 8	91 ± 10	<0.001
Age, yrs	46.0 ± 11.5	45.3 ± 12.6	47.0 ± 9.8	0.505
Male	31 (41.3)	19 (42.2)	12 (40.0)	0.840
BMI, kg/m^2^	24.5 [22.6, 27.1]	24.4 [22.8, 27.6]	24.7 [22.0, 26.6]	0.368
Cardiovascular risk factors				
Hypertension	10 (13.3)	6 (13.3)	4 (13.3)	0.885
Diabetes mellitus	3 (4.0)	1 (2.2)	2 (6.7)	0.560
Hypercholesterolaemia	10 (13.3)	6 (13.3)	4 (13.3)	1.000
Family history of heart disease	13 (17.3)	5 (11.1)	8 (26.7)	0.152
Smoking	3 (4.0)	3 (6.7)	0 (0.0)	0.270
Chronic renal insufficiency	3 (4.0)	2 (4.4)	1 (3.3)	0.810
Cardiovascular medications				
Beta-blocker	6 (8.0)	5 (11.1)	1 (3.3)	0.392
Non-dihydropyridine CCB	3 (4)	2 (4.4)	1 (3.3)	0.810
ACE inhibitor	4 (5.3)	2 (4.4)	2 (6.7)	0.675
Diuretic	5 (6.7)	4 (8.9)	1 (3.3)	0.642
Statin	6 (8.0)	3 (6.7)	3 (10.0)	0.678
Age at time of HL diagnosis, yrs	24.0 [19.0, 29.5]	25.0 [19.0, 32.0]	23.5 [21.0, 26.8]	0.721
Interval from HL therapy, yrs	17.6 [12.4, 25.8]	15.5 [12.1, 23.7]	23.6 [12.8, 26.9]	0.119
Ann Arbor stage III-IV	21 (28.0)	15 (33.3)	6 (20.0)	0.319
Radiotherapy type				0.061
Mantle field & subtotal nodalirradiation*	33 (44.0)	16 (35.6)	17 (56.7)	
IFRT including mediastinal irradiation	39 (52.0)	28 (62.2)	11 (36.7)	
IFRT without mediastinal irradiation	3 (4.0)	1 (2.2)	2 (6.7)	
Cumulative mediastinal radiation dose, Gy	36 [35,40]	36 [30,40]	36 [30,36]	0.659

Variable	Overall	Heart rate < 80 bpm.	Heart rate ≥ 80 bpm.	
	**(N = 75)**	**(N = 45)**	**(N = 30)**	***p*-value**
Chemotherapy regimen				0.668
ABVD*	21 (35.0)	12 (33.3)	9 (37.5)	
MOPP/ABV*	17 (28.3)	11 (30.6)	6 (25.0)	
EBVP*	7 (11.7)	3 (8.3)	4 (16.7)	
BEACOPP*	3 (5.0)	1 (2.8)	2 (8.3)	
MOPP	6 (10.0)	5 (13.9)	1 (4.2)	
Other	6 (10.0)	4 (11.1)	2 (8.3)	
Cumulative anthracycline dose, mg/m^2^	210 [150, 300]	205 [150, 285]	210 [150, 300]	0.583

Mean ± standard deviation, median [25th, 75th percentile], count (%); ACE = angiotensin-converting enzyme; CCB = calcium channel blocker; IFRT = involved field radiotherapy; HL = Hodgkin lymphoma; *other radiotherapy types did not include mediastinum.* both techniques always include mediastinal irradiation.

*chemotherapy regimen contains anthracycline

The average RHR in the study population was 77 ± 14 bpm with 30 patients (40%) having an elevated RHR (91 ± 10 vs. 69 ± bpm, *p*=<0.001) . There were no significant differences in baseline characteristics between patients with and without elevated RHR. Furthermore, patients with concomitant anthracycline-containing chemotherapy did not have a significantly increased RHR compared to patients whom received only thoracic irradiation (80 ± 11 vs. 77 ± 15; *p =* 0.453).

A summary of echocardiographic parameters of systolic and diastolic function is presented in Table 2. While LVEF was similar in patients with and without elevated RHR, GLS was significantly lower in patients with an elevated RHR (−15.9% vs. −18.3%, *p =* 0.045).

**Table 2:** Echocardiographic characteristics of the total population and according to the presence of an elevated resting heart rate.

Variable	Overall	Heart rate < 80 bpm.	Heart rate ≥ 80 bpm.	
	**(N = 75)**	**(N = 45)**	**(N = 30)**	***p*-value**

LVESVi, ml/m^2^	21.1 ± 6.0	21.0 ± 10.8	21.4 ± 6.7	0.762
LVEDVi, ml/m^2^	45.5 ± 11.3	47.1 ± 10.4	43.0 ± 12.3	0.124
Biplane LVEF, %	55.3 ± 5.3	55.6 ± 4.3	54.8 ± 6.6	0.543
E/A	1.0 [0.8, 1.2]	1.1 [0.9, 1.3]	0.9 [0.8, 1]	0.013
e' (average), cm/s	12.5 [10.5, 15]	13.0 [11.0, 17.0]	10.8 [10.0, 13.2]	0.038
E/e'	5.6 [4.6, 7.5]	5.3 [4.6, 6.4]	6.6 [4.8, 8.7]	0.034
LAVi, ml/m^2^	19.5 ± 5.2	20.2 ± 4.7	18.4 ± 5.7	0.139
GLS, %	−17.8 [-19.3, −15.7]	−18.3 [-19.3, −17.1]	−15.9 [-19.7, −14.3]	0.045
Abnormal GLS (>-16%)	24 (32.4)	8 (17.8)	16 (55.2)	0.002
Abnormal diastolic function	43 (57.3)	21 (46.7)	22 (73.3)	0.022

GLS = global longitudinal strain; LAVi = left atrial volume index; LVESVi = left ventricular end-systolic volume index; LVEDVi = left ventricular end-systolic volume index; LVEF = left ventricular ejection fraction

**Fig. 2:**
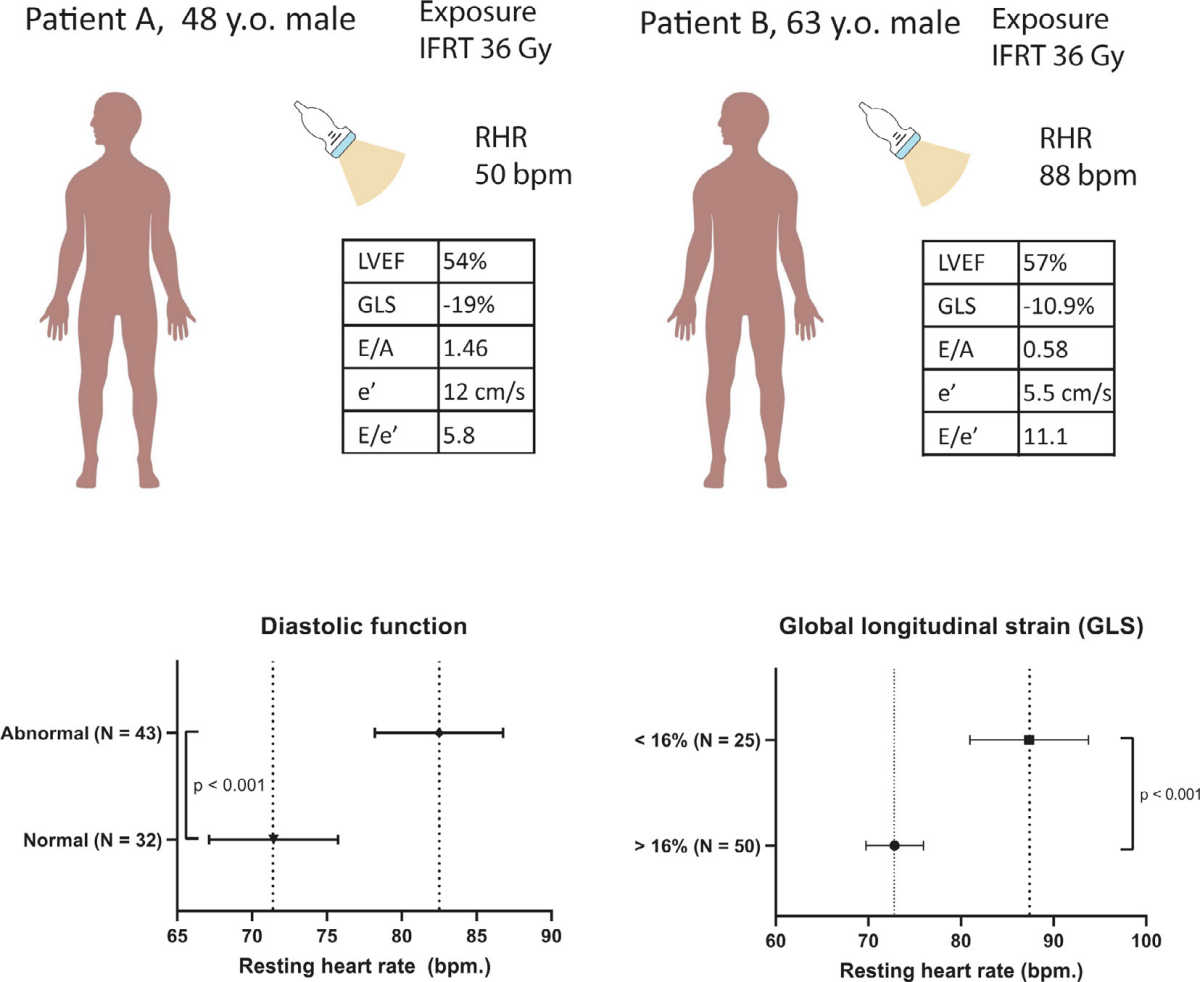
Example of two patients where (top) during echocardiographic examination, which shows that patient B has an increased RHR. (middle) Strain imaging analysis in addition to conventional markers of systolic and diastolic LV function reveal that patient B has both systolic and diastolic LV dysfunction. (below) Mean and 95% CI of resting heart rate in the study population by diastolic function (left) and global longitudinal strain (right).

Furthermore, presence of diastolic dysfunction was more prevalent among patients with elevated RHR as compared to patients without an elevated RHR: 73.3% vs. 46.7% (*p* = 0.022). This is emphasized by a significantly decreased e’ wave velocity (*Asymp. p* = 0.038), and increased E/e’ ratio - which has been well correlated with LV filling pressure (*Asymp. p =* 0.034) in patients with elevated RHR [27]. Moreover the presence of diastolic dysfunction was associated with higher RHR: 82 ± 14 bpm vs. 71 ± 12 in absence of diastolic dysfunction; *see* Fig. 2. Also, abnormal GLS was associated with higher RHR (83 ± 14 vs. 71 ± 12, *p* < 0.001).

Noteworthy, left atrial volume in the total population was low: 19.5 ± 5.2 mL/m^2^. Due to this lack of atrial dilation, it was not feasible to further classify diastolic dysfunction from grade I to grade III, as atrial dilation is required by the aforementioned guidelines [24].

In multilinear regression analysis GLS (per %), E/e’ ratio (per point) and presence of diastolic dysfunction were independently associated with RHR when controlling for age, sex and mantle field irradiation: see Table 3. Per % reduction in GLS, RHR increased with 1.30 bpm (95% CI: 0.12 – 2.48; *p* = 0.031) and in case of diastolic dysfunction RHR increased with 8.38 bpm (95% CI: 1.50–15.26, *p =* 0.018).

**Table 3:** Multilinear association model of echocardiographic parameters of left ventricular function with resting heart rate.

Determinant		B	95% for B	P-value
GLS, per %	Unadjusted	2.23	1.16 – 3.31	<0.001
	Adjusted*	1.81	0.69 – 2.92	0.002
E/e', per point	Unadjusted	1.94	0.81 – 3.07	0.001
	Adjusted*	1.29	0.03 – 2.54	0.045
Abnormal diastolic function	Unadjusted	11.05	4.95–17.15	0.001
	Adjusted*	8.38	1.50 – 15.26	0.018*

mean ± standard deviation, median [25th, 75th percentile], count (%); GLS = global longitudinal strain

When we compared an extracardiac confounder model (age + sex + mantle field irradiation) with an unrestricted model that included the before-mentioned confounders (GLS and diastolic dysfunction, we observed a significant improvement in association with RHR [F-statistic = 6.36, *p =* 0.003)]. This further supports our hypothesis that LV function is associated with RHR.

## 4 Discussion

The present study reports the result of comprehensive echocardiographic examinations in HL survivors treated with thoracic irradiation to assess the association of LV function and the presence of an elevated RHR.

Key findings of the study are (i) systolic- and diastolic dysfunction are more prevalent among patients with an elevated RHR, and (ii) both GLS and diastolic dysfunction are independently associated with RHR. To our knowledge this is the first study to demonstrate an association between RHR and subclinical LV dysfunction in cancer survivors. The results confirm earlier studies showing high prevalence of both systolic and diastolic dysfunction in patients treated with thoracic irradiation and/or anthracyclines [18], [19], [20], [21], [28], [29], [30], [31].

RHR is the product of a balance between vagal and sympathetic stimulation of the sino-atrial node. Increased adrenergic drive with positive chronotropic effects is one of the key mechanism in early stages of heart failure [22]. While at first increased RHR acts as a compensatory mechanisms to restore cardiac output, it later becomes detrimental to LV function as heart failure progresses. For this reason an elevated RHR in cancer survivors treated with radiation- and/or chemotherapy might reflect subclinical LV dysfunction and indicate worse long-term prognosis [9], [15], [32], [33].

Moreover, while LVESVi remained similar between both strata of RHR, there was a trend towards smaller LVEDVi in patients with an elevated RHR. As previously described, this reduction in stroke volume could be the consequence of myocardial fibrosis and LVEDVi shrinkage associated with cardiotoxic cancer treatment. *Izzo et al.* demonstrated the inverse correlation with stroke volume changes and plasma norepinephrine (NE) concentrations in 68 mild hypertensive patients [34]. Possibly, elevated RHR in long term HL survivors could be the result of these increased plasma levels of NE. *Grassi et al*. displayed that elevated RHR can be considered a marker of sympathetic hyperactivity in both the general population and patients with cardiovascular disease. However, its value for this purpose is limited and the relation was most evident in heart failure and obese patients. In contrast, patients with essential hypertension did not show a correlation between elevated RHR and indices of sympathetic hyperactivity. While the HL survivors in this study do not have clinical heart failure, their background is also different from that of the general population or patients with cardiovascular disease because they have chemotherapy- and/or radiation induced myocardial fibrosis and impaired diastolic function [35]. The value of resting heart rate in this specific population should be further investigated.

Accordingly, several studies focused on whether RHR could serve as a therapeutic target to reduce cardiovascular events and mortality aside of patients heart failure with reduced ejection fraction, where heart rate reduction is a cornerstone of therapy. In cancer biology, sympathetic nervous system activation has been known to promote tumour growth and metastasis, and consequently a multitude of observational epidemiological studies have been conducted to assess the favourable effect of betablockers in a variety of solid tumours [36], [37], [38]. Several studies showed a minor benefit in overall survival, but this has not been supported by clinical trials and meta-analyses reported conflicting results [39]. Carvedilol has been studied in small clinical trials for the prevention of anthracycline-induced cardiotoxicity in breast cancer patients and did show preservation of LV function compared to placebo, but well-powered randomised controlled trials with clinical endpoints have yet to be performed [40], [41]. Physical exercise in cancer survivors is known to lower RHR and in several studies lower RHR has been associated with lower all-cause mortality and therefore serves as an interesting target for supportive care during and after cancer treatment [42], [43], [44].

Both systolic and diastolic dysfunction were more prevalent among patients with an elevated RHR and importantly abnormal GLS, increased E/e’-ratio and any grade of diastolic dysfunction were independently associated with RHR. The relation between RHR and LV function was underlined by a significant improvement of the association model between RHR and extracardiac determinants – age, sex and use of mantle field irradiation – when echocardiographic parameters of LV function were included (e.g. GLS and presence of diastolic dysfunction). These findings suggest that an elevated RHR in these patients might not be a harmless sign, but a potential marker for underlying LV dysfunction as a result of anti-cancer treatment. It must be noted, while GLS and E/e’ were significantly more abnormal in patients with an elevated RHR, the observed systolic and diastolic dysfunction was subtle and the clinical implication of this subtle dysfunction needs to be further investigated. However, these findings were observed during the first echocardiographic examination ten years after mediastinal irradiation in an overall young population. It is likely that systolic and diastolic dysfunction will become increasingly more important during longer follow-up as several publications have shown that compared to the general population, HL patients have a significantly increased risk of cardiovascular disease [45], [46]. Further studies are needed to evaluate serial changes in left ventricular function during longer follow-up and the relation with adverse outcome.

Another notable finding is that in patients with diastolic dysfunction left atrial volume was low. Increase in left atrial size is usually one of the first signs of increased LV filling pressure and is therefore incorporated in classification algorithms of diastolic function [24]. Because there was no substantial atrial dilation in our cohort, diastolic dysfunction was identified through tissue doppler imaging and further grading of diastolic function was not possible. A possible explanation could be that mediastinal irradiation also leads to fibrosis and scarring of the atria [29], [47], [48]. Diastolic dysfunction induced by mediastinal irradiation therefore has a different pathogenesis and left atrial size in this population may remain normal due to the fibrosis that leads to atrial stiffening and less atrial dilatation. The average E/A was 1.0 in the study population, which suggest another possible explanation for the absence of left atrial enlargement: a possible large proportion of patients having early (grade I) diastolic dysfunction, where left atrial enlargement is less frequent.

Thoracic irradiation leads to a broad range of cardiac complications - including coronary artery disease, radiation valvulopathy, pericarditis and heart failure - through damaging both cancer- and healthy cells by creating reactive oxygen species that induce local inflammation and tissue fibrosis [3]. Anthracyclines are frequently combined with irradiation therapy and have the potential to induce dose-dependent cardiomyopathy by causing irreversible oxidative damage to cardiac mitochondria resulting in apoptosis [49], [50]. The increased risk of adverse cardiac events extends beyond forty years after treatment in HL survivors [2]. While there are clear childhood cancer survivorship guidelines, post-treatment surveillance for cardiac adverse effects in (young) adult cancer survivors has been troublesome [6], [51] and additional indicators of occult late-cardiotoxicity are needed. Our findings suggest that an elevated RHR is a potential marker for early myocardial dysfunction and could be helpful to define patients at risk for adverse cardiac events. More research is needed to elaborate on the clinical implications of elevated RHR and subclinical LV dysfunction in cancer survivors to further improve risk stratification and guide long term follow up.

In our study a cut-off value of 80 bpm was used to define elevated RHR. Over the years both higher and lower thresholds have been utilized. While dichotomization of RHR - as being elevated or not - is helpful for conceptualization purposes, a “true” RHR is substantially influenced by testing circumstances, measuring method and medication. For instance, some patients used betablockers or non-dihydropyridine calcium channel blockers which have negative chronotropic properties and could lower RHR. While in regression analysis betablocker use and RHR were not independently associated, our study population was too small to study the effect of these medications on RHR. Furthermore, while RHR was determined when a stable heart rate had established, it is possible that some misclassification in RHR strata can occur as a result of physical discomfort by the ultrasound probe.

To objectively assess LV function, patients with acute coronary syndrome or cardiac surgery have been excluded from our study populations, therefore the implication of these results cannot be extrapolated to all HL survivors. Furthermore, there is little spread in cumulative mediastinal irradiation dose in the study population and for this reason it is not possible to draw conclusions on dose-dependent occurrence of subclinical LV dysfunction or presence of an elevated RHR, but earlier studies have already supported this principle [52].

## 5 Conclusion

The results of our study indicate that elevated RHR is a common finding in HL survivors treated with thoracic irradiation. Importantly, elevated RHR was associated with LV diastolic dysfunction and subclinical systolic dysfunction as assessed by GLS.

## Funding

The department of Cardiology received research grants from Biotronik, Medtronic and Boston Scientific.
